# *MDR1* gene polymorphisms are associated with ulcerative colitis in a cohort of Serbian patients with inflammatory bowel disease

**DOI:** 10.1371/journal.pone.0194536

**Published:** 2018-03-15

**Authors:** Dragana Mijac, Irena Vukovic-Petrovic, Vera Mijac, Vladimir Perovic, Natasa Milic, Srdjan Djuranovic, Daniela Bojic, Dragan Popovic, Djordje Culafic, Miodrag Krstic, Goran Jankovic, Vera Pravica, Milos Markovic

**Affiliations:** 1 Clinic for Gastroenterology and Hepatology, Faculty of Medicine, University of Belgrade, Clinical Center of Serbia, Belgrade, Serbia; 2 Institute of Microbiology and Immunology, Faculty of Medicine, University of Belgrade, Belgrade, Serbia; 3 Department for Medical Statistics and Informatics, Faculty of Medicine, University of Belgrade, Belgrade, Serbia; 4 Department of Internal Medicine, Mayo Clinic, Rochester, MN, United States of America; 5 Department of Gastroenterology, University Hospital Zvezdara, Faculty of Medicine, University of Belgrade, Belgrade, Serbia; University Hospital Llandough, UNITED KINGDOM

## Abstract

**Background:**

Inflammatory bowel disease (IBD) is a chronic disease of unknown etiology in which genetic factors contribute to development of disease. Single nucleotide polymorphisms (SNPs) in multidrug resistance 1 (*MDR1*) gene encoding transporter P-glycoprotein have been associated with IBD, but their role in disease susceptibility remains unclear. Therefore, the aim of this study was to investigate the association of three *MDR1* polymorphisms, C1236T (rs1128503), G2677T/A (rs2032582) and C3435T (rs1045642), with Serbian IBD patients.

**Methods:**

A total of 206 IBD patients, 107 Crohn's disease (CD) and 99 ulcerative colitis (UC), and 255 healthy controls were included in the study. All subjects were genotyped using TaqMan SNP genotyping assays. Comparisons between the groups were performed using the Pearson Chi-square test. False discovery rate according to Benjamini-Hochberg procedure was applied to adjust for multiple comparisons.

**Results:**

Carriers of T allele of all three *MDR1* SNPs were more common in UC patients compared to healthy controls, suggesting predisposing role of T allele of these SNPs in UC pathogenesis. Consistently, TT genotype of C1236T and TTT haplotype were also found more frequently in UC patients. On the other hand, C allele and CC genotype of C1236T and C3435T, as well as G allele and GG genotype of G2677T/A were more frequent in healthy subjects, implying protective role of these variants in UC. Likewise, CGC haplotype and CGC/CGC diplotype were more frequent in controls. Contrary to UC, no statistical difference was observed between CD patients and controls in any of the SNPs analyzed.

**Conclusion:**

*MDR1* gene variants and haplotypes were associated with UC in Serbian IBD patients, further supporting their potential role in susceptibility to UC.

## Introduction

Inflammatory bowel disease (IBD) with its major forms, ulcerative colitis (UC) and Crohn's disease (CD), is a chronic, relapsing, lifelong disorder of the gastrointestinal (GI) tract. IBD occurs worldwide and due to the increase in its incidence noted in the last 50 years it is becoming an important global health problem [1]. Despite extensive studies, the etiopathogenesis of IBD is still not fully understood. However, evidence suggests that it is multifactorial and comprises aberrant immune response to microbiota on mucosal barrier of GI in genetically susceptible individuals [2]. Linkage studies in families clearly emphasize the importance of genetic factors to disease development. Between 5 and 23% of patients with IBD have an affected first-degree relative, with up to 25% chance that families with CD have members with UC and vice versa, which is consistent with the hypothesis that some risk gene alleles are common to both disorders, while other susceptibility genes may be unique specifically either to UC or CD [3, 4].

Advances in genetic methodology in the last decades, from candidate gene approach to genome-wide association studies (GWAS), have led to identification of many genes that could be related to IBD [4]. One of the candidate genes that got a lot of attention is *ABCB1* (ATP-binding cassette, subfamily B 1 gene), also called multidrug resistance 1 (*MDR1*) gene. *MDR1* encodes P-glycoprotein 170 (P-gp), an ATP-dependent drug transport efflux pump, highly expressed in many cells and epithelial surfaces, including the epithelium of GI tract [5]. In the gut, P-gp is expressed constitutively on the apical surfaces of the superficial epithelial cell layer, with the lower levels of expression in stomach, and gradual increase from duodenum towards ileum and distal parts of intestine [6]. The physiological function of P-gp has not been elucidated thus far. However, it is known that substrates for the P-gp pump include a variety of structurally and pharmacologically distinct hydrophobic compounds, such as toxins and drugs. Pgp could act as a protective barrier to keep toxins out of the body by secreting them into bile, urine, and intestinal lumen, and thereby preventing their accumulation in critical organs [7, 8]. It might also play a critical role in host-bacterial interactions in the gastrointestinal tract and maintenance of intestinal homeostasis. Therefore, it is possible that mutations of the *MDR1* gene could affect the uptake of bacterial toxins and xenobiotics and thus have a role in the development of IBD. In line with this notion are the results from the study with *mdr1a* knockout mice [9]. When kept in specific pathogen-free environment, those mice develop spontaneous colitis which resembles UC in humans, but shares some features of CD, as well. In humans, *MDR1* gene is contained within the region on chromosome 7q that has been linked with IBD [10]. Moreover, it was shown that *MDR1* expression is significantly reduced in colonic tissue of patients with UC [11].

More than 50 single-nucleotide polymorphisms (SNPs) of *MDR1* that occur naturally in humans have been identified thus far and some of them were related to altered P-gp expression and function [12, 13]. Three most common SNPs that have been repeatedly shown to predict changes in the function of P-gp are synonymous SNPs C1236T (rs1128503) in exon 12 and C3435T (rs1045642) in exon 26 and non-synonymous, triallelic SNP G2677T/A (Ala893Ser/Thr or rs2032582) located in exon 21 [14]. The association of C3435T and G2677T/A with IBD has been extensively studied in the past years. One of the first observations that both T allele and TT genotype of C3435T polymorphism were more frequently present among UC subjects came from a German study [15]. The positive association of G allele of 2677 and IBD was also shown in a large multicenter North American study [16]. However, a large number of later reports that analyzed different allele combinations of those SNPs in IBD patients gave conflicting results [7, 17–20]. Similarly to C3435T and G2677T/A, several studies has recently addressed the role of C1236T in IBD yielding also inconclusive results [20–25].

Considerable geographical and ethnical variations in IBD incidence have been demonstrated worldwide [26]. Likewise, *MDR1* expression and P-gp function also show wide inter-individual and interethnic differences, probably influenced by both environmental and genetic factors [27]. So far, significant interethnic differences in allele and genotype frequencies of *MDR1* SNPs have been reported, but most studies were done on populations from Western Europe, while data from Eastern European countries and Serbia in particular, are limited. Therefore, the aim of this study was to assess the three-locus genotype pattern for C1236T, G2677T/A and C3435T variants in Serbian patients with IBD.

## Material and methods

### Subjects

The study involved a total of 206 IBD patients and 255 unrelated healthy controls. IBD group comprised 107 CD patients and 99 UC patients followed up for IBD at Clinic for Gastroenterology and Hepatology, Faculty of Medicine, University Belgrade, Clinical Center of Serbia in the period from June 2012 to June 2014. Diagnosis of CD and UC was determined according to established guidelines based on standard clinical, radiological, endoscopic and histological criteria [28, 29]. Phenotype characteristics of UC and CD were defined according to the Montreal classification [30]. The clinical disease activity was calculated using the Mayo score and Crohn’s Disease Activity Index (CDAI) for UC and CD patients, respectively [31, 32]. Clinical characteristics of the patients included in the study are summarized in Table 1. Matching blood samples were obtained from 255 healthy blood donors using centralized procurement through the National Blood Transfusion Institute. Informed written consent was obtained from all individuals before blood sampling and the study was approved by the Ethic Committees of the Faculty of Medicine–University of Belgrade and Clinical Center of Serbia.

**Table 1:** Main clinical characteristics of CD and UC patients.

	CD	UC
Number	107	99
Sex–n (%)		
Male	62 (57.9)	54 (54.5)
Female	45 (42.1)	45 (45.5)
Age (years, median, min–max)	40 (18–68)	42 (19–70)
Age at diagnosis (years, median, min–max)	32.5 (14–67)	36.6 (2–67)
Disease duration (years, median, min–max)	6.6 (0–31)	5.5 (0–26)
Localisation–n (%)		
CD Ileal ± UGI	27 + 1 = 28 (26.2)	
Ileocolonic ± UGI	45 + 7 = 52 (48.6)	
Colonic ± UGI	27 + 0 = 27 (25.2)	
UGI (only)	0	
UC Proctitis		2 (2.0)
Left side colitis		35 (35.4)
Extensive colitis		59 (59.6)
IPAA-pouchitis		3 (3.0)
Behaviour–n (%)		
Inflammatory ± perianal	22 + 12 = 34 (31.7)	
Stricturing ± perianal	42 + 15 = 57 (53.3)	
Penetrating ± perianal	11 + 5 = 16 (15.0)	
Perianal (only)	0	
Perianal (any)	32 (29.9)	
Extra-intestinal manifestations–n (%)		
Current	18 (16.8)	27 (27.3)
Previous	16 (15.0)	12 (12.1)
Never	73 (68.2)	60 (60.6)
Activity index (median, min–max)	CDAI 151 (44–400)	Mayo 6 (0–12)
Early disease (up to 18 months)–n (%)	40 (37.4)	45 (45.5)
Surgery–n (%)	53 (49.5)	3 (3)
Age at Surgery (years, median, min–max)	34.7 (19–66)	48.7 (30–65)
Disease duration to surgery (years, median)	2.9	2.3
Corticosteroid-dependent–n (%)	54 (50.5)	32 (32.3)
Corticosteroid-refractory–n (%)	14 (13.1)	4 (4.0)
Anti-TNF therapy		
Yes	42 (39.3)	7 (7.1)
No	65 (60.7)	92 (92.9)
Smoking–n (%)		
At diagnosis	25 (23.4)	10 (10.1)
Ex smoker	11 (10.3)	19 (19.2)
Never	71 (66.3)	70 (70.7)

UGI–upper gastrointestinal; IPAA–ileo-pouch anal anastomosis; CDAI–Crohn's disease activity index; TNF–Tumor necrosis factor

### Genotyping

Genomic DNA was extracted from peripheral blood using the GeneJET whole blood genomic DNA purification mini kit (Fermentas Thermo Fisher Scientific Inc, Germany). The concentration and purity of DNA were determined by measuring absorbance at 260 nm and 280 nm. Detection of *MDR1* SNPs were done by real-time PCR using commercial primers and TaqMan probes (C_7586662_10 for C1236T, C_11711720C_30 and C_11711720D_40 for G2677T/A and C_7586657_20 for C3435T, all from Applied Biosystems Inc, USA) with Maxima Probe qPCR Master Mix (Fermentas Thermo Fisher Scientific Inc) following procedure recommended by the manufacturer of oligonucleotide mixes.

### Statistical analysis

Statistical analyses were performed using the statistical package for social sciences, version 20 (SPSS, Chicago, USA). The estimation of haplotypes and diplotype frequencies was done by the Expectation-Maximization algorithm using Arlequin 3.5.1.3 [33]. Comparisons between allele, genotype, haplotype and diplotype frequencies in different populations were performed using the Pearson Chi-square test. Two-sided p values, odds ratios (OR) and 95% confidence intervals (CI) were calculated. False discovery rate according to Benjamini-Hochberg procedure was applied to adjust for multiple comparisons. Calculations for the critical value were done with a false discovery rate of 5%.

## Results

Genotypes of C1236T, G2677T/A and C3453T SNPs were in Hardy-Weinberg equilibrium in the control group, as well as in all cases (CD and UC groups). The distribution of alleles and genotypes for C3435T, G2677T/A and C1236T in our study population is presented in Table 2. Overall, the frequencies of both alleles and genotypes of all three investigated *MDR1* SNPs were significantly different in UC patients compared to healthy subjects, whereas no statistical difference was observed between CD and controls in any of the SNPs analyzed (Table 2).

**Table 2:** Allele and genotype frequencies of *MDR1* SNPs in controls, CD and UC groups.

*MDR1* SNP		Controls (n = 255)	CD (n = 107)		UC (n = 99)	
		n (%)	n (%)	p value*	n (%)	p value*
**C1236T (rs1128503)**						
Allele	C	285 (55.9)	126 (58.9)	0.458	81 (40.9)	0.0003**
	T	225 (44.1)	88 (41.1)		117 (59.1)	
Genotype	CC	85 (33.3)	38 (35.5)	0.708	16 (16.2)	0.002**
	CT	115 (45.1)	50 (46.7)		49 (49.5)	
	TT	55 (21.6)	19 (17.8)		34 (34.3)	
**G2677T/A (rs2032582)**						
Allele	G	282 (55.3)	125 (58.4)	0.616	84 (42.4)	0.004**
	T	221 (43.3)	85 (39.7)		113 (57.1)	
	A	7 (1.4)	4 (1.9)		1 (0.5)	
Genotype	GG	83 (32.5)	35 (32.7)	0.674	16 (16.2)	0.007**
	GT	112 (43.9)	52 (48.6)		51 (51.5)	
	GA	4 (1.6)	3 (2.8)		1 (1.0)	
	TT	53 (20.8)	16 (15.0)		31 (31.3)	
	TA	3 (1.2)	1 (0.9)		0 (0)	
	AA	0 (0)	0 (0)		0 (0)	
**C3435T (rs1045642)**						
Allele	C	262 (51.4)	94 (43.9)	0.067	79 (39.9)	0.006**
	T	248 (48.6)	120 (56.1)		119 (60.1)	
Genotype	CC	70 (27.5)	33 (30.8)	0.449	13 (13.1)	0.013**
	CT	122 (47.8)	54 (50.5)		53 (53.5)	
	TT	63 (24.7)	20 (18.7)		33 (33.3)	

*p values were calculated using Chi-square test

**significant after adjustment for multiple test comparison

Regarding the C1236T SNP, minor T allele and TT genotype of C1236T were more frequent in UC patients compared to healthy controls (59.1% vs. 44.1%; p = 0.0003, OR = 1.830, 95%CI [1.312–2.552] for T allele and 34.3% vs. 21.6%; p = 0.013, OR = 1.902 [1.141–3.171] for TT genotype, respectively), suggesting predisposing role of T allele in UC. Conversely, CC genotype of C1236T could be recognized as a possible protective factor since it was significantly more common among healthy subjects then in UC patients (33.3% vs. 16.2%; p = 0.001, OR = 0.386 [0.213–0.699]). Likewise, T allele of C3435T was more common in UC patients compared to controls (60.1% vs. 48.6%; p = 0.006, OR = 1.426 [1.022–1.990]), whereas the frequency of CC genotype was significantly lower in UC patients than in controls (13.1% vs. 27.5%; p = 0.004, OR = 0.4 [0.210–0.761]). The allele and genotype distributions of triallelic G2677T/A SNP also differed significantly between UC patients and healthy subjects implying that allele T could be considered as a risk factor and G allele as protective factor in UC. Namely, T allele and TT genotype were more frequent in UC patients (57.1% vs. 43.3%; p = 0.001, OR = 1.739 [1.248–2.422] for T allele and 31.3% vs. 20.8%; p = 0.037, OR 1.738 [1.032–2.927] for TT genotype, respectively), although the significance for TT genotype was lost after the correction for multiple testing. On the other hand, G allele and GG genotype had higher frequency in control group (55.3% vs. 42.4%; p = 0.002, OR = 0.596 [0.428–0.830] for G allele and 32.5% vs. 16.2%; p = 0.002, OR 0.4 [0.220–0.725] for GG genotype, respectively). In contrast to UC, no significant differences in allele or genotype frequencies of C1236T, G2677T/A and C3435T were observed in CD patients in comparison to the control group.

Furthermore, we compared the frequencies of allele carriers of C1236T, G2677T/A and C3453T in controls and UC group (Table 3). Carriers of A allele of G2677T/A SNP were excluded from the analysis since only one UC patient had that allele. As a result, we found that the carriers of T allele of all three SNPs were more frequent in UC patients than in healthy controls suggesting predisposing role of T variants of these SNPs in UC pathogenesis in our patients. Consistently, carriers of presumably protective C allele of C1236T were also found to be significantly more common in control group than in UC patients.

**Table 3:** Comparison between allele carriers of *MDR1* SNPs in controls and UC group *MDR1* SNP.

	Controls (n = 255)	UC (n = 99)		
	n	n	p value*	Odds ratio (95% CI)
**C1236T (rs1128503)**				
C carrier	200	65	0.013**	0.526 (0.315–0.876)
T carrier	170	83	0.001**	2.594 (1.430–4.703)
**G2677T/A (rs2032582)**				
G carrier	199	68	0.067	0.617 (0.368–1.036)
T carrier	168	82	0.002**	2.498 (1.394–4.475)
**C3435T (rs1045642)**				
C carrier	192	66	0.101	0.656 (0.396–1.088)
T carrier	185	86	0.004**	2.503 (1.314–4.770)

*p values were calculated using Chi-square test

**significant after adjustment for multiple test comparison

CI–confidence interval

Finally, we determined distribution of three-locus (1236, 2677 and 3453) haplotypes and diplotypes in our study groups. A total of 10 haplotypes and 20 diplotypes were identified, but only three haplotypes (CGC, CGT and TTT) and six diplotypes (CGC/CGC, CGC/CGT, CGC/TTT, CGT/TTT, TTC/TTT and TTT/TTT) had frequencies higher than 5% and were included in the analysis (Table 4). CGC haplotype and CGC/CGC diplotype were found more common in healthy controls than in UC patients (46.9% vs. 34.3%; p = 0.003, OR = 0.593 [0.422–0.834] for CGC haplotype and 22.7% vs. 11.1%; p = 0.013, OR = 0.425 [0.213–0.848] for CGC/CGC diplotype, respectively) further validating potential protective role of 1236C, 2677G and 3453C alleles in UC. Similarly, TTT haplotype was significantly more frequent among UC patients (54% vs. 40.8%; p = 0.001, OR = 1.707 [1.226–2.376]), which was in concordance with allele/genotype/carrier analyses recognizing T alleles of all three *MDR1* SNPs as risk factors in UC. Consistent with this finding, we observed higher frequency of TTT/TTT diplotype in UC group compared to controls (26.3% vs. 18.8%), but the difference did not reach statistical significance (p = 0.122). On the other hand, TTT in conjunction with rare TTC haplotype (TTC/TTT) was associated with UC (p = 0.028, OR 4.468 [1.047–19.364]), but this nominal association did not withstand correction for multiple testing. Again, no significant difference in haplotype/diplotype distribution was recorded in CD in comparison to the control group.

**Table 4:** Haplotype and diplotype frequencies of *MDR1* SNPs in controls, CD and UC groups.

*MDR1* Haplotype/Diplotype	Controls (n = 255)	CD (n = 107)		UC (n = 99)	
	n (%)	n (%)	p value*	n (%)	p value*
**Haplotype**					
C G C	239 (46.9)	107 (50.0)	0.441	68 (34.3)	0.003**
C G T	30 (5.9)	14 (6.5)	0.735	12 (6.1)	0.928
T T T	208 (40.8)	78 (36.4)	0.276	107 (54.0)	0.001**
Other	33 (6.5)	15 (7.0)		11 (5.6)	** **
**Diplotype**					
C G C / C G C	58 (22.7)	26 (24.3)	0.752	11 (11.1)	0.013**
C G C / C G T	15 (5.9)	8 (7.5)	0.570	2 (2.0)	0.127
C G C / T T T	89 (34.9)	39 (36.4)	0.777	42 (42.4)	0.188
C G T / T T T	11 (4.3)	6 (5.6)	0.596	5 (5.1)	0.512
T T C / T T T	3 (1.2)	2 (1.9)	0.606	5 (5.1)	**0.028**
T T T / T T T	48 (18.8)	14 (13.1)	0.186	26 (26.3)	0.122
Other	31 (12.2)	12 (11.2)		8 (8.1)	** **

*p values were calculated using Chi-square test

**significant after adjustment for multiple test comparison

## Discussion

The present study is the first genetic association study that investigated allele and genotype patterns of three common polymorphisms in the *MDR1* gene (C1236T, G2677A/T and C3453T) in IBD patients from Serbia. Its results demonstrated a highly significant association of all three investigated SNPs with an overall susceptibility for UC. Moreover, we also observed significant difference in the haplotype and diplotype distributions between UC patients and healthy controls. In contrast, none of the analyzed SNPs was associated with CD patients in our cohort.

There is a great degree of controversy on the contribution of SNPs in the *MDR1* gene to IBD susceptibility. Regarding the C3435T and G2677T/A variants, the first published studies revealed significant association with UC and IBD in Whites from Germany and USA, respectively [15, 16], but subsequent studies only partially replicated the initial findings since they have produced contradictory results (Table 5). As for C3435T polymorphism, some studies found association of T allele and/or TT genotype with UC and not with CD, in German, Scottish, Japanese, Iranian and Croatian populations [15, 34–38], whereas many studies failed to demonstrate these associations in different populations [16, 17, 20–25, 39–51]. Rare data pointed even to the association of C3435T polymorphism with CD [52, 53]. Despite such discrepancy in results from different studies, it seems that mutant T allele of C3435T could be regarded as a risk factor for UC, since several meta-analyses confirmed the association of this variant with susceptibility to develop UC, but not CD [7, 17, 18, 20]. In our study, T allele of C3435T was clearly associated with UC which is in line with its presumed pathogenic role in the pathogenesis of this form of IBD. Similarly to C3435T, the association between triallelic G2677T/A SNP and IBD was extensively investigated with inconsistent results (Table 5). In our study, T allele was associated with UC patients, while wild G allele and GG genotype dominated within control group, implying predisposing and protective roles of T and G variants, respectively. Our carrier analysis further supported possible pathogenic role of T allele in UC. Similar associations of T allele with UC were observed in British, Indian, Croatian and Slovenian studies [17, 23, 38, 40]. In contrast, G allele was associated with IBD as a risk factor in American patients [16] and as a protective factor for CD in Chinese study [25]. A number of studies failed to demonstrate any association of G2677T/A and UC or CD patients [35, 36, 42, 45–49, 53], indicating that this polymorphism of *MDR1* may not confer susceptibility to IBD, as suggested by the negative results of two meta-analyses performed thus far [7, 18]. In contrast to C3435T and G2677T/A, C1236T polymorphism has been less extensively analyzed with regard to IBD (Table 5). The strongest association observed in our study was the association between T allele of C1236T and UC. The frequency TT genotype was also significantly higher in UC patients, while C allele and CC genotype were more common in healthy controls, suggesting predisposing and protective role of T and C allele, respectively. Again, our carrier analysis further substantiated these findings. Allele T or TT genotype of C1236T SNP have been already linked to UC susceptibility in Moroccan and North Indian IBD patients [20, 23], while C allele and CC genotype were associated with UC patients in China [24]. On the other hand, several studies found no association between C1236T and UC or CD [21, 25, 36, 40, 43] and two recent meta-analyses also failed to demonstrate such associations [18, 20].

**Table 5:** Summary of *MDR1* association studies and meta-analyses in IBD.

Study	Ref.	Population	Number of cases/studies		Analyzed SNPs	Main results
**Association studies**						
Schwab et al. 2003	15	German	CD 126	UC 149	C3435T	3435T allele associated with UC
Brant et al. 2003	16	American	CD 409	UC 119	C3435T, G2677T/A	G2677 allele associated with IBD
Croucher et al. 2003	39	German and British	CD 562	UC 307	C3435T	No association
Glas et al. 2004	34	German	CD 135	UC 123	C3435T	Association of 3435T allele with UC depending on the control group used
Potocnik et al. 2004	40	Slovenian	CD 163	UC 144	C3435T, G2677T/A C1236T	2677T allele associated with UC TTT haplotype associated with UC
Gazouli et al. 2004	41	Greek	CD 120	UC 85	C3435T	No association
Palmieri et al. 2005	42	Italian	CD 478	UC 468	C3435T, G2677T/A	No association
Ho et al. 2005	35	Scottish	CD 268	UC 335	C3435T, G2677T/A	T allele and TT genotype of C3435T and G2677/3435T haplotype associated with UC
Ho et al. 2006	21	Scottish	CD 179	UC 249	C3435T, C1236T	No association
Osuga et al. 2006	36	Japanese	CD /	UC 66	C3435T, G2677T/A C1236T	3435T allele associated with later onset UC
Onnie et al. 2006	17	British	CD 828	UC 580	C3435T, G2677T/A	2677T associated with UC
Urcelay et al. 2006	53	Spanish	CD 321	UC 330	C3435T, G2677T/A	C allele and CC genotype of C3435T and 2677T/C3435 haplotype associated with CD
Oostenbrug et al. 2006	43	Dutch	CD 553	UC 224	C3435T, C1236T	No association
Lal et al. 2006	52	Canadian	CD 247	UC 112	C3435T	3435T allele associated with CD
Lee et al. 2006	44	Korean	CD 24	UC 94	C3435T	No association
Fiedler et al. 2007	45	German	CD 244	UC 144	C3435T, G2677T/A	No association
Fischer et al. 2007	46	Hungarian	CD 265	UC 149	C3435T, G2677T/A	No association
Farnood et al. 2007	37	Iranian	CD /	UC 300	C3435T	T allele, TT and CT genotypes of C3435T associated with UC
Ardizzone et al. 2007	47	Italian	CD 211	UC 97	C3435T, G2677T/A	No association
Sapmaz et al. 2008	48	Turkish	CD 35	UC 82	G2677T/A	No association
Huebner et al. 2009	22	New Zealand	CD 383	UC 401	C3435T, G2677T/A C1236T	G2677T and C1236T heterozygous protective for UC
Krupoves et al. 2009	54	Canadian	CD 270 (childr.)	UC /	C3435T, G2677T/A C1236T	No association
Juyal et al. 2009	23	Indian	CD /	UC 270	C3435T, G2677T/A C1236T	1236T allele and TTT, TGT and 1236T/2677T haplotypes associated with UC
Ostergaard et al. 2009	49	Danish	CD 373	UC 541	C3435T, G2677T/A	No association
Dudarewicz et al. 2012	50	Polish	CD 47	UC 61	C3435T	No association
Brinar et al. 2013	38	Croatian	CD 199	UC 109	C3435T, G2677T/A	T allele of G2677T/A, TT genotype of C3435T and 2677T /3435T haplotype associated with UC Heterozygous C3435T protective for CD
Bonyadi et al. 2014	51	Iranian Azeri Turks	CD 19	UC 97	C3435T	No association
Cao et al. 2015	24	Chinese	CD /	UC 61	C3435T, C1236T	C allele and CC genotype of C1236T associated with UC
Senhaji et al. 2015	20	Moroccan	CD 77	UC 33	C3435T, C1236T	TT genotype of C1236T associated with UC
Yang et al. 2015	25	Chinese	CD 117	UC 39	C3435T, G2677T/A C1236T	G2677 protective for CD
**Meta-analyses**						
Annese et al. 2006	7	Various	7 studies		C3435T, G2677T/A	T allele and TT genotype of C3435T associated with UC No association for G2677T/A
Onnie et al. 2006	17	Various	7 studies		C3435T	3435T associated with UC
Zintzaras et al. 2012	18	Various	18 studies		C3435T, G2677T/A C1236T	3435T associated with UC No association for G2677T/A and C1236T
Wang et al. 2014	19	Various	13 studies		C3435T	No association
Senhaji et al. 2015	20	Various	18 studies		C3435T, C1236T	3435T associated with IBD No association for C1236T

In complex diseases, like IBD, haplotype analysis is potentially more informative than analysis of single polymorphisms, as it takes into account the combined effects of all SNPs tested and any variation within a region that might be in the linkage disequilibrium (LD) with them. Therefore, we determined whether three-locus (1236, 2677, 3435) haplotypes provide a stronger association with disease than individual SNPs in our cohort. As a result, both susceptible (TTT) and protective (CGC) haplotypes for UC were identified that can alter the risk for developing disease in a bidirectional fashion. The putative susceptible haplotype, which was strongly associated with our UC patients, contained mutant allelic variants (T variants) of all three SNPs that are believed to increase the risk to develop UC. On the other hand, the supposed protective haplotype that prevailed among healthy subjects in our cohort included wildtype alleles (C1236, G2677 and C3435) which were in previous studies occasionally shown to lower the risk for the disease. In line with this, CGC/CGC diplotype was also associated with UC patients in our study. There are a limited number of studies that assessed the influence of three-locus (1236, 2677, 3435) haplotypes on IBD susceptibility and they found association between TTT and UC [23, 40]. Likewise, some two-locus haplotypes (namely, 1236T/2677T, G2677/3435T and 2677T/3435T) were also associated with UC [23, 35, 38, 45]. Finally, some studies did not find any association between haplotypes and susceptibility to develop UC [22, 25, 54]. Thus, our haplotype data are in accordance with our results from analyses of single polymorphisms as well as with results from most other studies, further validating important role of certain variants of C1236T, G2677T/A and C3435T SNPs in determining susceptibility to UC, but not CD.

Apparently, there is uncertainty concerning the role of C1236T, G2677T/A and C3435T polymorphisms in IBD pathogenesis due to conflicting results of the studies in different populations. The reasons for reported differences may be relatively small sample size in most studies leading to a lack of association due to low power, differences in study design and selection of control population. The importance of controls has already been shown in German study in which the association depended on the control group chosen by the investigators [34]. The discrepancy can also be explained by ethnic diversity. There is some evidence that the polymorphisms allele frequencies may be different in subpopulations and that the frequency of minor mutant alleles of *MDR1* SNPs varies considerably between different populations (e.g. about 3% for C3435T compared to online hapmap database in Caucasians) [7, 38, 55]. Since the differences in *MDR1* SNPs observed between patients and healthy subjects are quite modest, it is possible that the minor allele frequencies in the control groups account for the majority of different results in various studies [38]. In our study, the frequencies of alleles in control group were comparable to other European studies, especially those from the region where populations are believed to share the same origin [38, 40, 55]. Our findings of significant associations of C1236T, G2677T/A and C3435T SNPs with UC are in line with possible predisposing role of T variants of these polymorphisms in disease pathogenesis. However, it is not clear how those variants may confer susceptibility towards UC and why no such predisposition was observed in CD. Likely explanation would be that altered P-gp expression and/or function caused by *MDR1* SNPs may result in an aberrant transport of potentially toxic xenobiotics and/or microbial products in individuals who harbor particular variant(s) making them more susceptible to develop UC, but not CD. The G2677T is a coding polymorphism resulting in substitution of alanine by serine and 2677T allele was shown to increase P-gp function [56], although other studies found no influence of this SNP on expression level, intracellular location or function of P-gp [57–59]. On the other hand, C1236T and C3435T are silent polymorphisms that do not cause change in amino acid sequence of P-gp. Nevertheless, C3435T SNP was associated with altered P-gp expression and function in some studies [12, 60], but other studies failed to confirm that [59, 61]. As for C1236T, no report has investigated so far whether this polymorphism may be functional. Despite limited and contradictory results, it is possible that all three polymorphisms (or at least some of them) may impact *MDR1* gene expression and P-gp function, either alone or synergistically, as suggested by the association of TGT and TTT haplotypes with the function of P-gp [56, 62]. However, it cannot be excluded that some other unidentified causal variant (or variants) that is in LD with the analyzed *MDR1* SNPs, affects expression and/or function of P-gp and thus confers susceptibility to develop UC.

The main question that is remaining is why highly powered GWAS have not found evidence for *MDR1* polymorphism associations seen in candidate gene studies [63]. There are several possible explanations for the observed discrepancy. First, it is simply possible that *MDR1* polymorphism associations have not been evaluated properly, as it might be the case for the triallelic variant (G2677T/A polymorphism). Second, GWAS are designed to screen the genome, based on the LD between genotyped SNPs and the potentially causative variants, so it is possible that *MDR1* polymorphisms have been missed because they have not been tagged by the genotyped SNPs or they may be located considerably far away from SNPs showing the disease association with UC. Next, because of the stringent significance thresholds due to multiple testing corrections, GWAS have less power than more confined studies, such are candidate gene studies, to detect genes with small effects on disease development, which may lead to a lack of associations of *MDR1* with UC in GWAS. Further, it is believed that mutations in *MDR1* could affect the uptake of bacterial toxins and xenobiotics, which may add an element of exposure to a complexity of UC pathogenesis, similarly to infectious diseases. Thus, certain variants might only be detectable in patients exposed to some (un)known environmental factors. Likewise, other factors, including IBD subphenotypes and interethnic differences of the populations studied may also affect results from various GWAS, as previously mentioned. A recent GWAS performed in African Americans with IBD has identified loci associated with UC only in this population, in addition to loci identified previously in European populations, further emphasizing the importance of the population included in the study [64]. On the other hand, it is possible that high-powered studies did not show evidence simply because there is no association of *MDR1* polymorphisms with UC. Or it may be that the negative candidate gene studies have not been published enough or corrections for multiple testing in published studies have not been done leading to false positive results. It is also possible that the candidate gene studies, which do not genetically match controls rigorously as GWAS, may be tending to report only stratification in allele frequencies between cases and controls. It is anticipated that the use of integrated analysis of genome (whole genome sequencing) and expression data (whole exome sequencing) will overcome some of the limitations of both types of genetic studies, which might confirm associations of some variants of MDR1 with UC in the near future.

Finally, it is important to underscore some limitations of this study. Our study group was relatively small. Nevertheless, the power of our study was fairly adequate as it ranged from 49.6% for C3435T, and 64.6% for G2677T/A, up to 71.9% for C1236T. Also, all IBD patients were from the tertiary referral center, and thus most of them had severe forms of disease. Consequently, patients with limited disease with low activity are underrepresented in our study. Thus, like in all association studies on complex diseases, replication of the associations in larger studies in ethnically similar populations is required to validate our findings.

## Conclusions

This is the first report on the prevalence of the *MDR1* polymorphisms in patients with IBD from Serbia, and one of the few performed so far in Eastern European countries. It is also one of the rare studies that included correction for multiple testing and still found associations of all three common *MDR1* polymorphisms (C1236T, G2677T/A and C3435T) with UC, with some of associations being among the strongest reported thus far with relatively high ORs, as for T allele of C1236T SNP. Moreover, susceptible and protective haplotypes for UC were also identified in our population. Therefore, our study may provide more insight into the complexities of the contribution of *MDR1* gene in determining susceptibility towards UC and add some valuable data in the search for reliable biomarkers in IBD.
